# Efficient conformational ensemble generation of protein-bound peptides

**DOI:** 10.1186/s13321-017-0246-7

**Published:** 2017-11-22

**Authors:** Yumeng Yan, Di Zhang, Sheng-You Huang

**Affiliations:** School of Physics, Huazhong University of Science and Technology, Wuhan, 430074 Hubei People’s Republic of China

**Keywords:** Conformer generation, Peptide, Molecular docking, Protein–peptide interactions, Conformational sampling

## Abstract

Conformation generation of protein-bound peptides is critical for the determination of protein–peptide complex structures. Despite significant progress in conformer generation of small molecules, few methods have been developed for modeling protein-bound peptide conformations. Here, we have developed a fast de novo peptide modeling algorithm, referred to as MODPEP, for conformational sampling of protein-bound peptides. Given a sequence, MODPEP builds the peptide 3D structure from scratch by assembling amino acids or helix fragments based on constructed rotamer and helix libraries. The MODPEP algorithm was tested on a diverse set of 910 experimentally determined protein-bound peptides with 3–30 amino acids from the PDB and obtained an average accuracy of 1.90 Å when 200 conformations were sampled for each peptide. On average, MODPEP obtained a success rate of 74.3% for all the 910 peptides and ≥ 90% for short peptides with 3–10 amino acids in reproducing experimental protein-bound structures. Comparative evaluations of MODPEP with three other conformer generation methods, PEP-FOLD3, RDKit, and Balloon, have also been performed in both accuracy and success rate. MODPEP is fast and can generate 100 conformations for less than one second. The fast MODPEP will be beneficial for large-scale de novo modeling and docking of peptides. The MODPEP program and libraries are available for download at http://huanglab.phys.hust.edu.cn/.

**Electronic supplementary material:**

The online version of this article (10.1186/s13321-017-0246-7) contains supplementary material, which is available to authorized users.

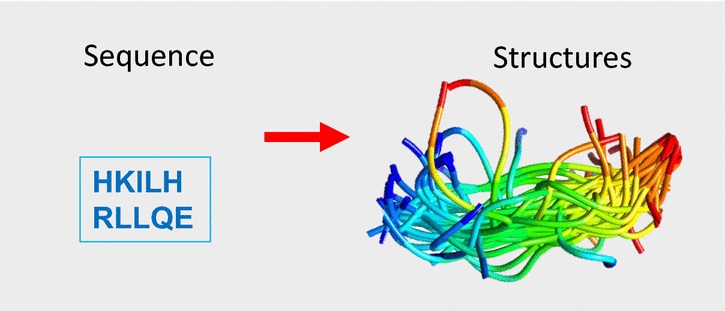

## Background

The interactions between peptides and proteins have received increasing attention in drug discovery because of their involvement in critical human diseases, such as cancer and infections [1–4]. It has been found that nearly 40% of protein–protein interactions are mediated by short peptides [2]. The biological function of a short peptide is related to its three-dimensional structure within its interacting protein. Therefore, determining the structures of protein–peptide interactions is valuable for studying their molecular mechanism and thus developing peptide drugs [5, 6]. However, due to the high cost and technical difficulties, only a small portion of protein–peptide complex structures were experimentally determined [7], compared to the huge number of peptides involved in cell function [8, 9]. As such, a variety of computational methods like molecular docking have been developed to predict the structures of protein–peptide complexes [3, 10–13].

Peptides are highly flexible and exist as an ensemble of conformations in solution. The biologically active conformation of a peptide is selected and/or induced when interacting with its protein partner. Therefore, a big challenge in protein–peptide docking is to consider the flexibility of peptides [12–16]. One way to consider peptide flexibility in docking is to fully sample the conformations of a peptide on-the-fly guided by its binding energy score [17–19]. However, given so many rotatable bonds in peptides, such sampling is computationally prohibitive. Therefore, current docking approaches often adopt a docking + MD protocol [20–22]. Nevertheless, this kind of docking + MD protocols is still computationally expensive and typically takes at least a few hours for docking a peptide [20–22]. Another way to consider peptide flexibility is through ensemble docking [23–25]. Namely, an ensemble of conformations for a peptide are first generated by a conformational sampling method and then docked against the protein by regular rigid docking [23]. A few top fits between the protein and the peptide conformations are selected as the predictions that may be subject to further refinement. Because of its high computational efficiency, ensemble docking has been widely used to consider molecular flexibility in both protein–protein and protein–ligand docking [10, 26, 27].

One critical part of ensemble docking is to generate an ensemble of peptide 3D models that include protein-bound peptide conformations, so that the biologically active ones can be selected by the protein during ensemble docking [3, 23, 28]. Despite significant progresses in the conformer generation of small molecules [29–36], few approaches have been developed for modeling of biologically active/protein-bound peptide conformations [37]. Therefore, a novel strategy is pressingly needed for efficient generation of protein-bound peptides. Meeting the need, we have developed a fast de novo approach for the generation of peptide 3D models, which is referred to as MODPEP. Instead of relying on a template, our MODPEP algorithm builds a peptide structure from scratch by assembling amino acids or helix fragments based on constructed rotamer and helix libraries. The peptide model building process is very fast and can generate a few hundred peptide conformations within seconds. Our method was validated on the peptide structures of 910 experimentally determined protein–peptide complexes from the protein data bank (PDB) [7].

## Methods

### Dataset compilation

**Fig. 1:**
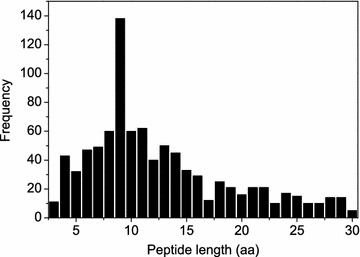
The observed frequencies of the peptides with different lengths in the test set, whose numbers are also shown in Tables 1, 2, 3 and 4

To construct rotamer libraries and validate our algorithm, we have developed a non-redundant dataset of experimentally determined protein-bound peptide structures. Specifically, we queried all the X-ray peptide structures in the PDB that met the following criteria. First, the peptide sequence contains at least three but less than 50 amino acids. Second, the structure has a resolution better than 3.0 Å. Third, the peptide does not contain non-standard amino acids. Fourth, the peptide must be bound to a protein. As of December 23, 2016, the query yielded a total of 3861 peptides meeting the above criteria. The sequences of the 3861 peptides were then clustered using the program CD-HIT [38]. If there are multiple peptide structures for a sequence, the structure with the highest resolution was selected to represent the sequence, resulting in a total of 2731 non-redundant peptide structures. It should be noted that unlike proteins which are often conserved in sequences, peptides often adopt a coil-like structure and are thus normally not conserved in sequences. Of these 2731 peptides, about two thirds (i.e. 1821) were randomly selected as the training database to construct the rotamer and helix libraries for peptide modeling, in which 878 peptides has a resolution between 2.0 and 3.0 Å. It should be noted that inclusion of the peptides with resolution of 2–3 Å should not have a significant influence on the backbone quality of the libraries and thus the prediction of peptide backbone, as according to X-ray crystallography, the positions of backbone and many side chains are clear in the electron density map at 2–3 Å resolution [39]. The rest 910 peptides were used as the test set to validate our algorithm. The frequencies of the peptides with different lengths are shown in Fig. 1 and Table 1.

**Table 1:** The average accuracies of our MODPEP method in reproducing protein-bound conformations for the peptides with different lengths when various ensemble sizes were considered

Peptide		cRMSD (Å)							
Length	Number	50	100	150	200	250	300	500	1000
3	11	0.05	0.04	0.04	0.04	0.04	0.04	0.03	0.03
4	43	0.27	0.23	0.22	0.21	0.20	0.19	0.16	0.14
5	32	0.59	0.50	0.47	0.45	0.43	0.40	0.36	0.33
6	47	0.96	0.78	0.71	0.65	0.64	0.61	0.55	0.52
7	49	1.23	1.08	0.98	0.96	0.93	0.88	0.79	0.72
8	60	1.70	1.52	1.35	1.29	1.27	1.23	1.16	1.02
9	138	1.89	1.71	1.63	1.56	1.52	1.48	1.38	1.26
10	60	1.97	1.81	1.73	1.67	1.61	1.56	1.52	1.39
11	62	2.33	2.20	2.08	2.04	2.00	1.98	1.88	1.70
12	40	2.43	2.25	2.15	2.09	2.06	2.03	1.85	1.71
13	50	2.66	2.43	2.40	2.29	2.25	2.22	2.11	1.95
14	45	3.05	2.89	2.75	2.64	2.60	2.56	2.44	2.28
15	33	2.84	2.68	2.60	2.58	2.56	2.56	2.47	2.35
16	29	2.95	2.75	2.71	2.66	2.61	2.55	2.48	2.38
17	12	2.95	2.72	2.66	2.56	2.53	2.50	2.37	2.31
18	25	3.13	3.03	2.98	2.93	2.91	2.85	2.74	2.55
19	21	2.66	2.61	2.46	2.38	2.30	2.29	2.17	2.03
20	16	3.52	3.28	3.25	3.14	3.07	3.04	2.92	2.85
21	21	3.47	3.24	3.11	3.05	2.99	2.98	2.88	2.74
22	21	2.70	2.54	2.44	2.43	2.42	2.39	2.35	2.23
23	10	3.32	3.27	3.07	3.01	3.00	3.00	2.78	2.74
24	17	3.37	3.24	3.13	3.11	3.08	3.03	2.92	2.74
25	15	2.93	2.78	2.72	2.71	2.62	2.51	2.40	2.32
26	10	3.06	2.97	2.89	2.77	2.73	2.72	2.67	2.62
27	10	4.35	4.14	4.12	3.95	3.89	3.89	3.69	3.51
28	14	3.32	3.16	3.10	3.05	2.96	2.93	2.87	2.68
29	14	4.63	4.45	4.31	4.24	4.24	4.08	3.98	3.76
30	5	2.90	2.87	2.82	2.72	2.72	2.71	2.64	2.64
All	910	2.20	2.04	1.95	1.90	1.86	1.83	1.73	1.62

### Rotamer library construction

**Fig. 2:**
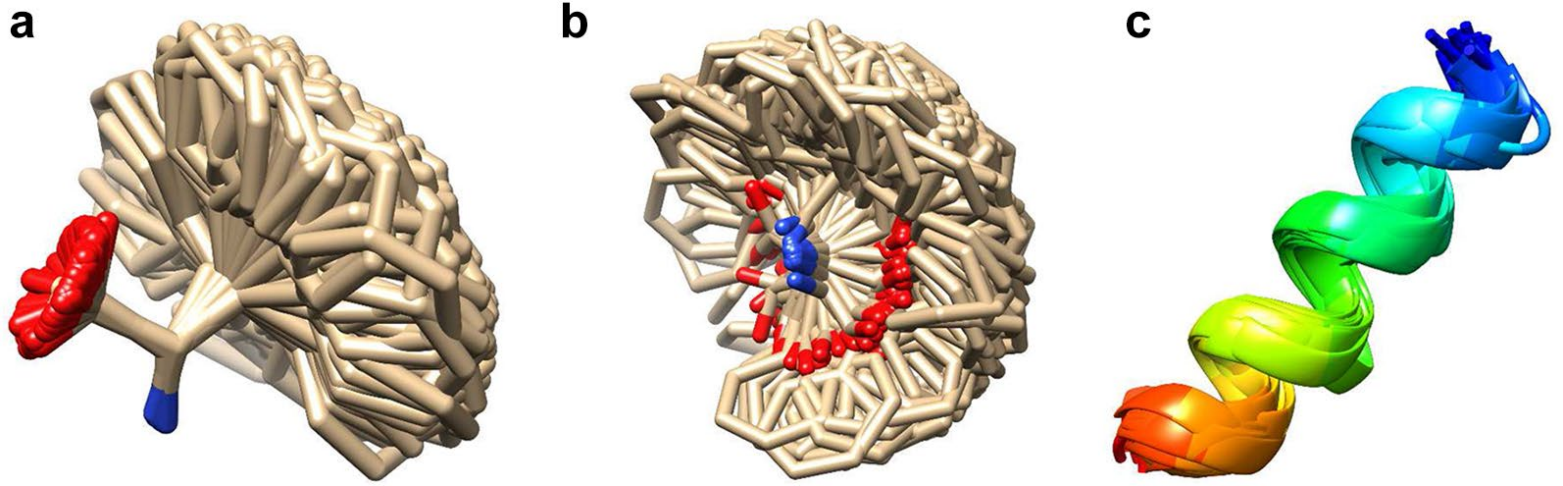
Examples of the **a** pure-rotamer and **b** C-rotamer libraries for amino acid PHE and **c** the helix fragment library with 16 amino acids

We have constructed two backbone-dependent rotamer libraries for peptide model building. The first library is called single-letter library, in which each rotamer consists of one amino acid residue (see Fig. 2a for an example). Therefore, we have a total of 20 single-letter libraries corresponding to 20 types of amino acids. They were used to build the side chain of an amino acid if only its backbone is available. Specifically, for each of the 20 amino acid types, all its residue conformations from the training database of 1821 peptides were aligned according to their N, CA, and C backbone atoms, and clustered using the root mean square deviation (RMSD) of all the heavy atoms of backbone and side chains. Two conformations were grouped into the same cluster if they have an RMSD of < 0.5 Å, resulting in multiple clusters for an amino acid type. For each cluster, the conformer including both backbone and side chain with the highest resolution was selected as a representative rotamer of the corresponding amino acid type. Dividing the number of conformations in a cluster by the total number of conformations for an amino acid type gives the probability of the rotamer for the amino acid type. The final number of conformers for an amino acid depends on its type. There are as few as six conformers for ALA and as many as 1075 conformers for ARG in the rotamer libraries.

The second rotamer library is a two-letter library, in which each rotamer is based on two consecutive amino acid residues (i.e. a dipeptide). The generating method for the two-letter library is similar to that for the one-letter library except for two aspects. One is that the rotamer for the two-letter library is based on dipeptides. For the first residue of a dipeptide conformation, only its backbone atoms (i.e. N, CA, C, O) was kept, which we call the HEAD of the dipeptide. The other is that the alignment between two dipeptide conformations is based on their HEAD atoms during the clustering. If two dipeptide conformations have an RMSD of less than 0.5 Å, they are grouped into the same cluster. For each cluster of a certain dipeptide type, the conformer with the higher resolution is selected as a representative rotamer of the two-letter or dipeptide type. Therefore, the rotamer in a two-letter library has one more HEAD than that in a single-letter library. Correspondingly, two-letter rotamers are more spread in space than single-letter rotamers (Fig. 2a, b). As the two-letter library constructed by this way is used to add a residue at the C-terminal of a peptide, we call it the C-rotamer library. Similarly, we have also constructed the N-rotamer library, in which the superimposition during clustering was based on the TAIL of dipeptides (i.e. the backbone atoms of the second residue).

### Helix library construction

In addition to rotamer libraries, we have also constructed a fragment library for helical structures with different lengths, where the secondary structure information was calculated using the program KSDSSP [40]. Because helix structures are relatively stable and do not much depend on sequences, we only kept the backbone atoms (i.e. N, CA, C, O) for the helix library. Side chains will only be added during model building, as described in the following section. Specifically, for a given peptide length, we have collected all the helix structures from the training database of 1821 peptides. All the helix conformations with the same length were then superimposed onto one another and clustered according to the RMSD of backbone atoms. If two helix conformations have an RMSD of less than 0.5 Å, they were grouped into the same cluster. It should be noted that the number of helical examples in the training set tended to be more limited for longer helices and thus resulted in fewer clusters. Depending on the lengths, the sizes of the libraries range from two clusters for the 28-residue helix to 37 clusters for the seven-residue helix. For each cluster of a helix length, the helix structure with the higher resolution was selected as a representative conformer of the helix length. For consistency, the backbone atoms (i.e. N, C, and CA) of the first residue of a helix fragment is called the HEAD of the helix, and the backbone atoms (i.e. N, C, and CA) of the last residue is called the TAIL of the helix fragment.

### Peptide structure modeling

With the constructed rotamer and helix libraries, our MODPEP algorithm can automatically build the three-dimensional structure of a peptide from scratch by assembling amino acids or helix fragments one by one. Specifically, given a peptide sequence, the program PSIPRED was first used to predict the second structure type (i.e. C-coil, S-sheet, or H-helix) of its amino acids [41]. Then, a rotamer was randomly selected from the single-letter library for the first amino acid of the sequence. If three or more consecutive amino acids including the current one on the sequence all had a secondary structure type of H-helix, a helix fragment was built by selecting a helix template from the helix library according to the probability of the helix structure and aligning the HEAD of the helix fragment with the corresponding backbone atoms of the current residue. The corresponding side chains for the helix fragment were built using the single-letter rotamer libraries according to the probability of its amino acid types. For all other cases that the next amino acid to be modeled has a secondary structure of C-coil or S-sheet type, the residue structure was stochastically built by selecting a rotamer from the C-rotamer library according to the probability of the rotamer and aligning the HEAD of the rotamer with the backbone of the current residue. The newly added amino acid or helix fragment was subject to an atomic clash checking. If there are severe clashes, the newly added rotamer or fragment will be discarded and a structure rebuilding process will be tried. The process was repeated until the last amino acid of the sequence was reached.

It should be noted that here the peptide 3D conformation of full length was built from N-terminal to C-terminal based on the C-rotamer and helix fragment libraries. However, the peptide structure can also be built from C-terminal to N-terminal by using the N-rotamer and helix fragment libraries. Our MODPEP algorithm can also construct the full peptide 3D structure for a partial one by building residues at both C-terminal and N-terminal. The peptide structure building process is very fast and can normally generate 100 peptide conformations in less than one second.

**Fig. 3:**
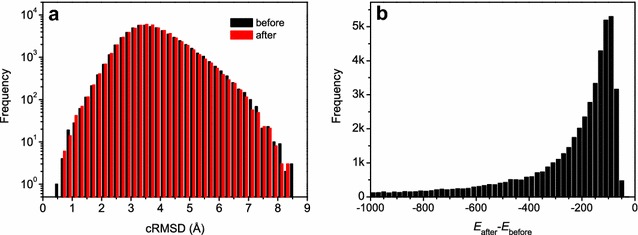
The accuracy distribution in terms of RMSD (**a**) and the energy difference ($\Delta E=E_{\mathrm{after}}-E_{\mathrm{before}}$) distribution (**b**) of the peptide models before and after minimization with AMBER for the peptides with 10 amino acids

For computational efficiency, we did not apply a complicated scoring function during model building and do an energy minimization for the generated models. Therefore, there might be a few bad bendings or torsional angles in the generated models. However, this does not affect the accuracy of the predicted models. As shown in a comparison between the original structures and the refined models by the ff14SB force field [42] of AMBER (version 14) [43], the refined ones are even slightly worse than the original models in terms of accuracy, although the refined models have a better energy scores than the original models (Fig. 3). The worse accuracy of the refined models compared to the original models can be understood because we are predicting the conformations of protein-bound peptides. The optimization of a peptide without its bound protein partner would drive the model further away from the protein-bound conformations, although the energy can also be minimized. Therefore, we have left the energy minimization of the generated models to users in real applications when they have a specific protein partner to be bound by the peptide.

### Evaluation criteria

The quality for a generated peptide model was measured by the root mean square deviation (RMSD) between the model and the experimentally determined peptide structures. Here, the RMSD was calculated based on the C*α* atoms of the peptide (cRMSD) after optimal superimposition of the two structures, as used in PEP-FOLD [44]. This is the default quality assessment parameter, unless otherwise specified. In addition, we have also calculated the RMSD of backbone heavy atoms (bRMSD) to evaluate the robustness of our approach and the RMSD of all heavy atoms (aRMSD) to check the capability of our method in predicting side chains.

For an ensemble of *N* conformations generated for a peptide, the accuracy of the ensemble was represented by the RMSD of the best-fit conformation in the ensemble compared to the experimentally observed structure. Therefore, a smaller RMSD means a higher accuracy. The accuracy depends on the number of considered conformations in the ensemble, i.e. the ensemble size.

It was found that a conformer with an RMSD of less than 1.0 Å was necessary for achieving a correct binding mode in molecular docking for compound ligands [45]. In other words, the generated conformer with an RMSD of less than 1.0 Å is similar to the experimental bound structure for short peptides from the perspective of chemistry. For medium-size peptides, an RMSD of less than 2.0 Å can be considered as native-like conformations [44]. In addition, RMSD is also size-dependent [46, 47], and larger proteins tend to give a larger RMSD for the similar accuracy [48]. Therefore, we have used a size-dependent RMSD cutoff as a criterion for successful predictions in the present study [48]

1 $$\begin{aligned} {\mathrm{rmsd}}_{\mathrm{C}}({\mathrm{n}})=1.0\times [1+\ln (n/n_0)] \end{aligned}$$

where *n* stands for the peptide length and $n_0$ was set as 3. The RMSD cutoff ranges from 1.0 Å for the peptides of 3 residues to 3.3 Å for the peptides of 30 residues. Thus, given a peptide of *n* residues, the peptide modeling was defined as a success if the accuracy of the ensemble is less than ${\mathrm{rmsd}}_{\mathrm{C}}({\mathrm{n}})$.

### Comparison with other methods

Comparing our MODPEP algorithm with other methods is difficult because few approaches have been developed for modeling protein-bound peptide structures, although there are published methods for conformational sampling of free peptides. Here, we have selected three state-of-art conformer generation algorithms, which are PEP-FOLD3 [49], RDKit (version 2016.09.4) [50], and Balloon (version 1.6.4.1258) [51], respectively. PEP-FOLD3 is a novel approach for de novo prediction of peptides and miniproteins. It assembles the peptide structure using a greedy procedure with Hidden Markov Model-derived structural alphabets [44]. RDKit adopts a distance geometry approach to generate conformers of a ligand. The resulting conformers were then optimized with the UFF force field [30, 52]. It was recently shown that RDKit was one of the best conformer ensemble generators on a high-quality benchmark of protein-bound ligand conformations [53]. Balloon is a method of conformer ensemble generation for ligands that aims to reproduce protein-bound ligand conformations [32]. It is also an implementation of distance geometry like RDKit. For both RDKit and Balloon, the code was downloaded from the authors’ web sites and evaluated locally. During the evaluation, the default parameters were used except that the number of conformers to be generated was set as 200. For PEP-FOLD3, because its code is not available for download, we obtained the test results by submitting the peptide sequences to the PEP-FOLD3 web server [37].

## Results and discussion

### Accuracy

With the constructed rotamer and helix libraries, we were able to model peptide structures using our fast MODPEP algorithm. The capacity of our peptide modeling algorithm in reproducing experimentally determined protein-bound conformations was evaluated on a test set of 910 peptides. For each peptide, we have generated an ensemble of 1000 conformations based on its sequence.

**Fig. 4:**
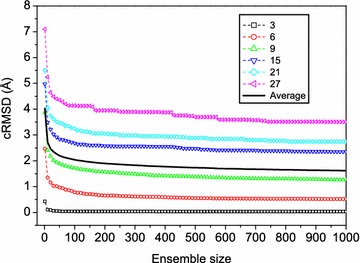
The average accuracies (bold solid line) of the best-fit predictions compared to the experimentally observed conformations as a function of ensemble size for the test set of 910 protein-bound peptides. For reference, the average accuracies for peptides of several typical lengths are shown

Figure 4 shows the average accuracy of our MODPEP in reproducing experimentally determined conformation as a function of ensemble size. The figure also shows the average accuracies of the peptides of six typical lengths (i.e. 3, 6, 9, 15, 21, and 27 amino acids). The detailed accuracies for several ensemble sizes are listed in Table 1. Several features can be observed from the figure and table. First, the accuracies depend on the peptide length. The shorter peptide gave a better accuracy with the lowest RMSD of 0.03 Å for 3-amino acid peptides and the highest RMSD of 3.76 Å for 29-amino acid peptides when an ensemble of 1000 conformations were considered (Table 1). Second, the accuracies also depend on the ensemble sizes of generated peptide conformations. Third, the accuracy is not a linear relationship with ensemble size. The accuracy changes faster at the beginning and then slower with the increasing number of conformations. On average, our MODPEP obtained an accuracy of 1.90 Å for an ensemble size of 200 and 1.62 Å for an ensemble size of 1000.

Figure 4 also shows that there roughly exists a crossover around 50 conformations on the accuracy-ensemble size curves for all peptide lengths. Therefore, an ensemble of 50 conformations for a peptide may be used if the computational resource is limited, though the accuracy always tends to be better for a larger ensemble size. Considering the accuracies for the peptides of all lengths, 200 conformations seem to be a good balance between the accuracy and the ensemble size (Fig. 4). Therefore, we have used 200 as the default ensemble size for our MODPEP algorithm in the following evaluations, though users can choose to generate more conformations in real applications. It can be observed from Table 1 that our MODPEP has an RMSD of 0.04 Å for the 3-amino acid peptide and an RMSD of 4.24 Å for the 29-amino acid peptide when the default ensemble size of 200 was used.

**Fig. 5:**
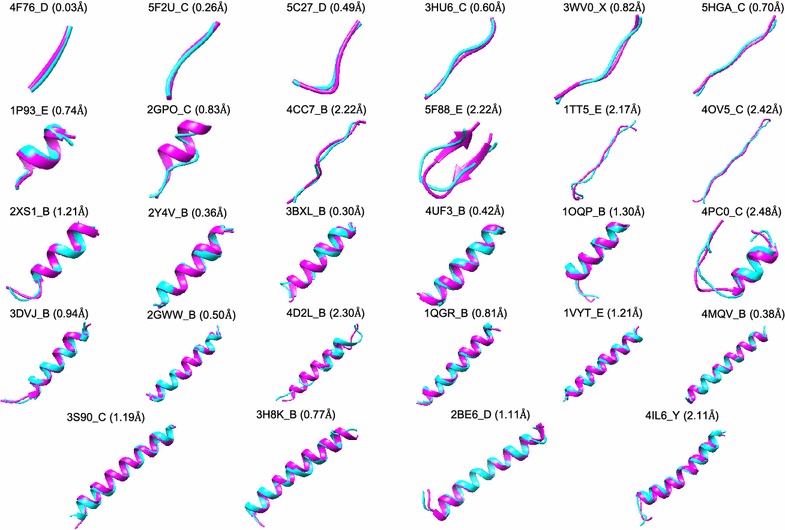
Examples of the predicted models for peptides with 3–30 amino acids, where each peptide is represented by its PDB code_chain ID. The native structure (magenta) is superimposed onto the predicted model (cyan). The corresponding accuracy is listed in parenthesis

**Fig. 6:**
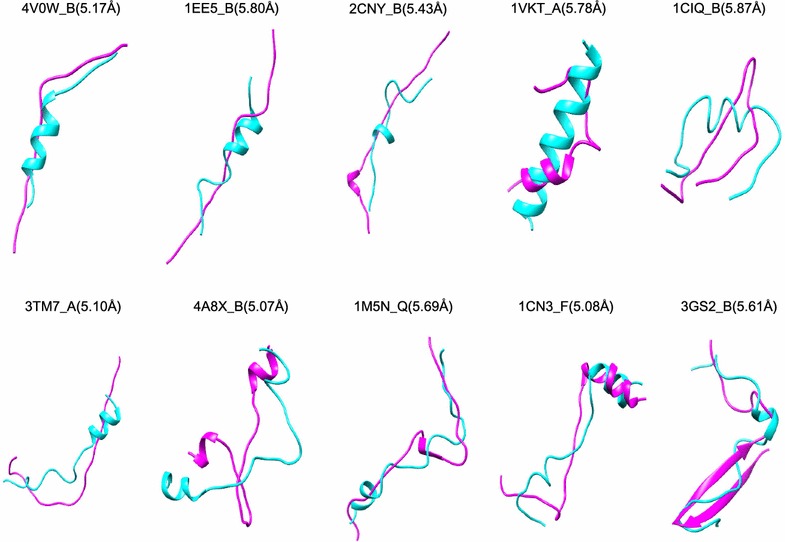
Examples of the predicted models for several challenging peptides, where each peptide is represented by its PDB code_chain ID. The native structure (magenta) is superimposed on the predicted model (cyan). The corresponding accuracy is listed in parenthesis

Figure 5 gives 28 examples of the predicted models with the RMSDs ranging from 0.03 to 2.48 Å for the peptides with 3–30 amino acids, respectively. It can be seen from the figure that the predicted models overlap with the experimental structures very well. Therefore, the present accuracy of MODPEP is good enough for direct docking calculations for peptides with 3–20 amino acids or provides a good starting point of docking + MD protocols for peptides with more than 20 amino acids. Nevertheless, MODPEP also failed to give models close to the experimental conformations for some peptides even when an ensemble of 1000 conformations were generated (Fig. 6). Several features can be found by examining these failed cases, which can help further improve our MODPEP algorithm. First, all the failed cases are medium or large-size peptides with more than 10 amino acids, as longer peptides tend to be more challenging to be predicted. Second, the secondary structures of some peptides are not correctly predicted by PSIPRED. Third, some peptides form a *β*-sheet structure with its protein partner. In such cases, it is challenging to generate correct *β*-sheet structure based on the peptide alone.

In addition, to check the statistical accuracy of MODPEP, we have repeated the validating procedure by splitting the data set into training and test sets for 10 runs. As shown in the Additional file 1, the prediction accuracies for different runs are quite consistent. On average, the standard deviations of the accuracies for 10 validating runs are around 0.02 Å for most peptide lengths, supporting the statistically robustness of MODPEP.

To further examine the robustness of MODPEP, we have also calculated the RMSD of generated peptide models based on the backbone and all the heavy atoms, respectively. Table 2 lists the average accuracies in terms of the RMSDs of C*α*, backbone, and all-heavy atoms for different peptide lengths when an ensemble of 200 conformations were considered. It can be seen from the table that the C*α* and backbone atoms yielded comparable RMSDs, while the all-heavy atoms gave a significant higher RMSD. This means that the higher RMSD of all-heavy atoms than backbone is due to side chains. The large RMSD induced by side chains can be understood as follows. First, although the backbone of protein is clearly visible in the electron density map at resolution of better than 3 Å, the accuracy of side chain positions significantly depends on the resolution [39]. Therefore, inclusion of side chains will not only impact the quality of the training set, but also the evaluation for the experimental peptide structures in the test set. Second, side chains tend to have larger induced conformational changes when a peptide binds to its protein partner. It is challenging to predict the positions of side chains without its bound protein. In other words, the conformations of side chains for a peptide are different depending on the protein that the peptide binds to. Namely, compared to the backbone, side chains are more binding-dependent and can only be correctly modeled upon binding. Therefore, we have used the C*α* RMSD as the default parameter to measure the accuracy of generated models in this study, as used in PEP-FOLD [44].

**Table 2:** The average accuracies of our MODPEP method measured using the C*α* (cRMSD), backbone (bRMSD), and all heavy atoms (aRMSD) for the peptides with different lengths when an ensemble of 200 conformations were considered for each peptide

Peptide		RMSD (Å)		
Length	Number	cRMSD	bRMSD	aRMSD
3	11	0.04	0.42	1.18
4	43	0.21	0.62	1.29
5	32	0.45	0.87	1.69
6	47	0.65	1.01	1.97
7	49	0.96	1.20	2.25
8	60	1.29	1.50	2.59
9	138	1.56	1.65	2.85
10	60	1.67	1.74	2.93
11	62	2.04	2.04	3.31
12	40	2.09	2.09	3.41
13	50	2.29	2.34	3.69
14	45	2.64	2.65	3.96
15	33	2.58	2.56	3.99
16	29	2.66	2.61	4.00
17	12	2.56	2.61	4.14
18	25	2.93	2.90	4.20
19	21	2.38	2.37	3.55
20	16	3.14	2.94	4.44
21	21	3.05	2.84	4.08
22	21	2.43	2.43	3.71
23	10	3.01	3.06	4.37
24	17	3.11	2.96	4.33
25	15	2.71	2.65	3.69
26	10	2.77	3.05	4.46
27	10	3.95	4.18	5.49
28	14	3.05	3.16	4.43
29	14	4.24	4.07	5.33
30	5	2.72	2.75	3.95
All	910	1.90	1.99	3.18

### Success rates

In addition to evaluating the accuracy of MODPEP, we have also calculated the success rate, i.e. the percentage of peptides in the test set that are successfully reproduced within the corresponding RMSD cutoff defined in Eq. 1. The corresponding results are shown in Table 3. It can be seen from the table that the success rates significantly depend on the peptide lengths. For example, for the peptides with 3–10 amino acids, MODPEP reproduced more than 95% of protein-bound peptide conformations when an ensemble of 200 models were considered (Table 3), while for the peptides with more than 10 amino acids, the success rates dropped below 80%. On average, our algorithm gave a success rate of 74.3% when an ensemble of 200 conformations were considered (Table 3).

**Table 3:** The success rates of our MODPEP method in reproducing protein-bound conformations for the peptides with different lengths when various ensemble sizes were considered

Peptide		Success rate (%)							
Length	Number	50	100	150	200	250	300	500	1000
3	11	100	100	100	100	100	100	100	100
4	43	100	100	100	100	100	100	100	100
5	32	100	100	100	100	100	100	100	100
6	47	97.9	97.9	97.9	97.9	97.9	97.9	97.9	97.9
7	49	98.0	100	100	100	100	100	100	100
8	60	83.3	91.7	93.3	95.0	96.7	96.7	96.7	96.7
9	138	75.4	92.8	96.4	97.8	98.6	98.6	98.6	99.3
10	60	71.7	81.7	90.0	96.7	96.7	98.3	98.3	98.3
11	62	45.2	58.1	69.4	77.4	79.0	80.6	82.3	90.3
12	40	37.5	42.5	47.5	52.5	55.0	57.5	80.0	92.5
13	50	38.0	48.0	50.0	58.0	62.0	64.0	74.0	80.0
14	45	31.1	33.3	42.2	48.9	51.1	53.3	62.2	80.0
15	33	30.3	36.4	36.4	36.4	36.4	36.4	42.4	57.6
16	29	27.6	34.5	34.5	41.4	44.8	44.8	48.3	51.7
17	12	41.7	41.7	41.7	50.0	50.0	50.0	58.3	58.3
18	25	36.0	40.0	40.0	44.0	44.0	44.0	48.0	52.0
19	21	57.1	61.9	61.9	61.9	66.7	66.7	66.7	71.4
20	16	43.8	50.0	50.0	50.0	50.0	50.0	50.0	50.0
21	21	33.3	42.9	42.9	42.9	42.9	47.6	57.1	57.1
22	21	57.1	61.9	61.9	61.9	61.9	61.9	61.9	71.4
23	10	50.0	50.0	50.0	50.0	50.0	50.0	50.0	50.0
24	17	41.2	41.2	47.1	47.1	47.1	47.1	47.1	52.9
25	15	60.0	60.0	60.0	60.0	60.0	60.0	66.7	66.7
26	10	40.0	40.0	40.0	40.0	40.0	40.0	40.0	40.0
27	10	20.0	30.0	30.0	30.0	30.0	30.0	30.0	30.0
28	14	35.7	42.9	42.9	42.9	50.0	50.0	50.0	57.1
29	14	21.4	21.4	21.4	21.4	21.4	21.4	21.4	28.6
30	5	60.0	60.0	60.0	60.0	60.0	60.0	60.0	60.0
All	910	61.6	68.7	71.5	74.3	75.4	76.0	79.0	82.9

**Fig. 7:**
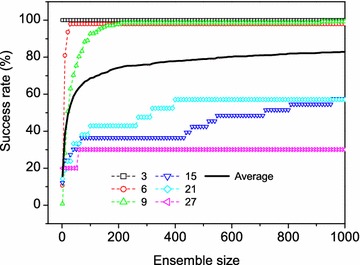
The success rates (bold solid lines) in reproducing experimentally determined protein-bound peptide conformations as a function of ensemble size. For reference, the results for the peptides of several lengths are shown

The success rates also depend on the ensemble sizes of generated conformations (Table 3). For example, for the peptides with 12 amino acids, the success rate in reproducing experimental structures is only 37.5% when an ensemble of 50 conformations were considered, but the success rate reached to 92.5% if an ensemble of 1000 conformations were considered (Table 3). The success rate also has a non-linear relationship with the ensemble size of generated conformations. The success rate increases fast at small ensemble sizes and become more stable at large ensemble sizes (Fig. 7). The algorithm achieved a good balance between the success rate and the ensemble size when 200 conformations were considered. With this ensemble size, peptides of most lengths have a success rate close to its maximum value (Table 3).

In addition, we have examined the impact of the secondary structure types on the quality of generated models. It was defined that if a peptide contained a *β*-sheet structure, it was characterized as the SHEET type; otherwise, it was classified as the HELIX type if the peptide contained a helix structure; the rest peptides belonged to the COIL type. Of 910 peptides in the test set, there are 304 peptides of HELIX type, 129 peptides of SHEET type, and 477 peptides of COIL type. MODPEP obtained a success rate of 83.6, 73.0, and 42.6% for the peptides of COIL, HELIX, and SHEET types, respectively, when an ensemble of 200 conformations were considered. This trend may be understood because MODPEP depends on the secondary structure information predicted by PSIPRED. Indeed, the accuracies of secondary structures prediction by PSIPRED showed a similar trend and had an average success rate of 85.1, 78.9, 53.5% for the secondary structures of COIL, HELIX, and SHEET types, respectively.

### Comparative evaluations

We further compared our MODPEP with three stat-of-art conformational sampling approaches, PEP-FOLD3, Balloon, and RDKit. It should be noted that PEP-FOLD3, Balloon, and RDKit are not designed for generation of protein-bound peptide conformations. Therefore, the present comparison is to provide a performance reference more than a comparative evaluation.

**Fig. 8:**
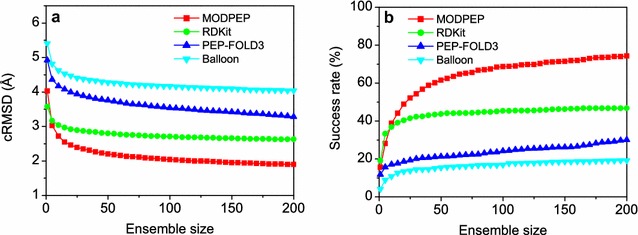
Comparison of the performances for four conformer generation methods, MODPEP, PEP-FOLD3, RDKit, and Balloon, on the test set of 910 protein-bound peptides. For each peptide, 200 conformers were generated per method. **a** Accuracy versus ensemble size, **b** success rate versus ensemble size

Figure 8 shows the average accuracy and success rate as a function of ensemble size by the four conformational sampling methods, MODPEP, PEP-FOLD3, RDKit, and Balloon, on the test set of 910 peptides. It can be seen from the figure that our method MODPEP obtained a much better performance than RDKit, PEP-FOLD3, and Balloon in terms of both accuracy and success rate. For example, MODPEP had an accuracy of 2.20, 2.04, and 1.90 Å, compared to 2.80, 2.71, and 2.63 Å for RDKit, 3.76, 3.54, and 3.28 Å for PEP-FOLD3, and 4.28, 4.17, and 4.04 Å for Balloon when ensembles of 50, 100, and 200 conformations were considered, respectively (Fig. 8a). Likewise, MODPEP reproduced the most protein-bound peptide conformations with an average success rate of 74.3%, followed by 46.8% for RDKit, 30.1% for PEP-FOLD3, and 19.2% for Balloon when an ensemble of 200 conformations were considered (Fig. 8b).

**Fig. 9:**
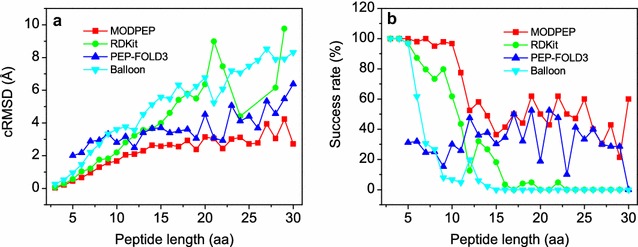
Comparison of the **a** average accuracies and **b** success rates of four conformer generation methods for peptides of different lengths when an ensemble of 200 conformations were considered

Table 4 and Fig. 9 show the average accuracies and success rates of MODPEP, RDKit, PEP-FOLD3, and Balloon for peptides with different lengths, respectively. Similar trends in the performances for the four methods can be observed in both accuracy and success rate. Namely, overall, MODPEP performed the best among the four methods, followed by RDKit, PEP-FOLD3, and Balloon. The relative performances of PEP-FOLD3 and RDKit/Balloon depended on the lengths of peptides. For short peptides with 3–8 amino acids, RDKit and Balloon performed better than PEP-FOLD3, while for longer peptides of more than 9 amino acids, PEP-FOLD3 performed better than RDKit and Balloon. For example, RDKit and Balloon had an average accuracy of 0.57 and 0.96 Å and a success rate of 100 and 100% for peptides of five amino acids, compared to 2.00 Å and 31.2% for PEP-FOLD3. However, for peptides with 17 amino acids, PEP-FOLD3 obtained an accuracy of 3.50 Å and a success rate of 50%, while RDKit and Balloon only had an accuracy of 6.33 and 5.41 Å and did not reproduce any correct conformations. These results indicate that short peptides with less than 9 amino acids behave more like ligands than proteins and therefore resulted in a fair performance for ligand conformer generator methods like RDKit and Balloon. In contrast, owing to our de novo strategy of residue assembling from the rotamer library, MODPEP can achieve good performances for peptides of all lengths (Table 4).

**Table 4:** The average accuracies and success rates of MODPEP, PEP-FOLD3, Balloon, and RDKit in reproducing protein-bound conformations for the peptides with different lengths when an ensemble of 200 conformations were considered for each peptide

Peptide		cRMSD (Å)				Success rate (%)			
Length	Number	MODPEP	PEP-FOLD3	Balloon	RDkit	MODPEP	PEP-FOLD3	Balloon	RDkit
3	11	0.04	0.00	0.27	0.07	100	0.0	100	100
4	43	0.21	0.00	0.53	0.28	100	0.0	100	100
5	32	0.45	2.00	0.96	0.57	100	31.2	96.9	96.9
6	47	0.65	2.18	1.47	1.03	97.9	31.9	61.7	87.2
7	49	0.96	2.88	2.21	1.21	100	24.5	30.6	79.6
8	60	1.29	2.94	2.70	1.75	95.0	25.0	26.7	73.3
9	138	1.56	3.32	3.34	1.82	97.8	15.2	8.0	79.7
10	60	1.67	2.80	3.61	2.20	96.7	30.0	6.7	61.7
11	62	2.04	3.17	3.78	2.81	77.4	25.8	4.8	43.5
12	40	2.09	2.48	3.57	3.22	52.5	47.5	20.0	12.5
13	50	2.29	3.40	4.53	3.56	58.0	36.0	6.0	32.0
14	45	2.64	3.66	5.13	3.67	48.9	37.8	2.2	26.7
15	33	2.58	3.71	5.59	3.99	36.4	30.3	0.0	18.2
16	29	2.66	3.39	5.49	4.61	41.4	34.5	0.0	3.4
17	12	2.56	3.50	6.33	5.41	50.0	50.0	0.0	0.0
18	25	2.93	3.61	5.73	5.78	44.0	32.0	0.0	4.0
19	21	2.38	3.05	6.24	5.49	61.9	52.4	0.0	4.8
20	16	3.14	4.51	6.78	6.37	50.0	18.8	0.0	0.0
21	21	3.05	3.09	5.24	8.99	42.9	52.4	0.0	0.0
22	21	2.43	2.92	6.09	7.46	61.9	47.6	0.0	4.8
23	10	3.01	5.05	7.20	0.00	50.0	10.0	0.0	0.0
24	17	3.11	4.12	7.07	4.41	47.1	41.2	0.0	0.0
25	15	2.71	4.41	7.48	0.00	60.0	33.3	0.0	0.0
26	10	2.77	3.68	7.84	0.00	40.0	40.0	0.0	0.0
27	10	3.95	5.33	8.53	0.00	30.0	30.0	0.0	0.0
28	14	3.05	4.57	7.89	6.15	42.9	28.6	0.0	0.0
29	14	4.24	5.47	7.92	9.77	21.4	28.6	0.0	0.0
30	5	2.72	6.38	8.32	0.00	60.0	0.0	0.0	0.0
All	910	1.90	3.28	4.04	2.63	74.3	30.1	19.2	46.8

## Conclusions

We have developed a novel peptide modeling algorithm, referred to as MODPEP, for fast conformational ensemble generation of protein-bound peptides. With constructed rotamer and helix libraries, our MODPEP algorithm builds the peptide 3D structure from scratch by assembling amino acids or helix fragments according to a given sequence. MODPEP is fast and can generated 100 peptide conformations for less than one second. The accuracy of MODPEP depended on the ensemble size of generated conformations and on average had an RMSD of 1.90 Å on a diverse test set of 910 protein-bound peptides with 3–30 amino acids when 200 conformations were considered for each peptide. On average, MODPEP obtained an average success rate of 74.3% in reproducing experimentally determined structures for all the 910 tested peptides and a success rate of > 95% for the short peptides with 3–10 amino acids. MODPEP was compared to three other three approaches, PEP-FOLD3, RDKit, and Balloon. It was found that MODPEP performed significantly better in both accuracy and success rate in reproducing protein-bound peptide conformations.

## Additional file

**Additional file 1.** The average accuracies and standard deviations of MODPEP for the peptides of 3–30 amino acids on ten randomly splitted training/test sets.
